# Strong association between vibration perception thresholds at low frequencies (4 and 8 Hz), neuropathic symptoms and diabetic foot ulcers

**DOI:** 10.1371/journal.pone.0212921

**Published:** 2019-02-28

**Authors:** Eero Lindholm, Magnus Löndahl, Katarina Fagher, Jan Apelqvist, Lars B. Dahlin

**Affiliations:** 1 Department of Clinical Sciences, Endocrinology, Lund University, Malmö, Sweden; 2 Department of Clinical Sciences, Endocrinology, Lund University, Lund, Sweden; 3 Department of Translational Medicine—Hand Surgery, Lund University, Malmö, Sweden; 4 Department of Hand Surgery, Skåne University Hospital, Malmö, Sweden; Baylor College of Medicine, UNITED STATES

## Abstract

**Aims:**

To investigate whether multi-frequency measurement of vibration perception thresholds (VPTs) can identify individuals with a high risk of developing diabetic foot ulcer or neuropathic symptoms.

**Methods:**

VPTs were measured at six different frequencies (4, 8, 16, 32, 64 and 125 Hz) on metatarsal heads 1 and 5 on the sole of the foot using a standard VibroSense Meter device in 535 type 1 diabetic (T1DM) patients and 717 non-diabetic control subjects. VPTs in control subjects were used to establish normal values for five different age groups for male and female subjects respectively. Normal values were defined as a VPT below the mean plus 1.66 x standard deviation for each group. Various definitions of abnormal VPTs were tested using either all frequencies, only lowest VPT frequencies (4 and 8 Hz) or only highest VPT frequencies (64 and 125 Hz).

**Results:**

The VPTs were higher in T1DM patients than in non-diabetic control subjects matched for age and gender. The low frequencies, 4 and 8 Hz, particularly were associated with the risk of diabetic foot ulcer (OR 40.7 [5.4–308.4], p = 0.0003) and with difficulties in balance and or gait (OR 1.89 [1.04–3.46], p = 0.04) difficulties and weakness (OR 2.77 [1.25–6.16], p = 0.01). The VPTs at the 125 Hz frequency were higher in short duration (≤ 10 yrs.) T1DM patients compared to age- and gender-matched control subjects.

**Conclusions:**

Vibration perception thresholds at low frequencies seem to be a better indicator of the risk of developing diabetic foot ulcers, gait or balance problems or weakness of the foot. The 125 Hz frequency, however, seemed to be impaired earlier and it was the only pathological VPT frequency in patients with short duration of diabetes.This study suggests that at least four different frequencies (4, 8, 64 and 125 Hz) should be included in any examination in order to obtain a complete evaluation of the risk factors for diabetic neuropathy and diabetic foot ulcers.

## Introduction

Diabetic neuropathy is one of the most important precipitating factors for diabetic foot ulcers [1]. Plantar foot ulcer constitute 22–25% of all foot ulcers and are usually localized at metatarsal heads or plantar surface of the first digit [2]. Estimates of the prevalence of diabetic neuropathy vary from 10 to 90% depending on the population and the criteria or methods are used [3]. Typically, neuropathy presents as sensory loss with neuropathic pain present in only 11 to 32% of the patients. Approximately half of the patients are asymptomatic [4]. Diabetic neuropathy is considered irreversible, possible because of the poor and insufficient axonal regeneration in diabetic patients.

Diabetic neuropathy can be assessed in several ways using different types of symptom scores and several kinds of measurements. The 128 Hz tuning fork and the 10 g monofilament test are widely used and recommended for screening for large fiber function and risk of developing diabetic foot ulcer and amputation [4]. Vibration perception thresholds seem to detect presymptomatic neuropathy earlier than the monofilament [5] Using 1 g monofilament could be more effective in early detection of diabetic polyneuropathy [6].

Vibrotactile sense is mediated by Pacinian corpuscles and Meissner´s corpuscles. It has been suggested that Pacinian corpuscles are most sensitive to frequencies in the region of 250–550 Hz [7], and Meissner´s corpuscles are most sensitive at 30 Hz [8, 9]. In clinical practice however, vibration perception thresholds are usually measured by biothesiometer/neurothesiometer; a hand-held device that uses only one frequency, usually 100–130 Hz. In this paper, such an instrument measuring VPT at only at one frequency is referred to as a biothesiometer regardless of the manufacturer. Patients with VPT > 25 V measured using a biothesiometer have been shown to have an increased risk of developing diabetic foot ulcer [10].

Multifrequency vibrometry is a method that has been used in clinical practice in occupational medicine to examine hand-arm vibration syndrome (HAVS) and has an excellent test-retest reliability [11]. The VPTs are measured at six different frequencies varying from 4 Hz up to 125 Hz (hand studies include 250 and 500 Hz). There is only one study on VPTs measured at several frequencies on the sole of the foot in adult type 1 and type 2 diabetic patients [12]. The study measured VPTs at three locations, at the metatarsal heads 1 (MTH 1) and 5 (MTH 5) and at the heel. The heel seemed to be less sensitive to vibration at least at 64 Hz frequency, and to perception of touch. VPTs were significantly higher in subjects with diabetes than in healthy subjects at low frequencies (8,16 and 32 Hz), but not at higher frequencies [12]. Although, the study population was small (N = 37), the results could indicate that the frequency currently used, 100–130 Hz, may not be the most sensitive for detecting neuropathy. Another study, examining VPTs in the fingers in TDM1 patients, shows increased VPTs, particularly at 250 and 500 Hz, in both the index and the little fingers, while the commonly used frequency 125 Hz shows no such increase[13]. Multifrequency vibrometry has also been studied in children and adolescents with type 1 diabetes, with a recent study by Ising et. al. showing that VPTs were already impaired in 18% of the children [14].

Vibration perception thresholds are affected by age and height [15] but this information has not been used in clinical practice. In this study, we have investigated the effects of age and gender on VPTs in non-diabetic controls to establish age- and gender-specific normal ranges for VPTs.

From the start studies on HAVS defined an abnormal VPT outcome as a sensibility index below 0.8 [16]. The sensibility index (SI) is a composite measure of all studied VPT frequencies. Therefore, when the sensibility index is used as a measure for an abnormal outcome some of the information content from measured frequencies is lost.

Studies using multifrequency vibrometry on diabetic patients have hitherto only compared VPTs in diabetic patients vs. non-diabetic control subjects without defining what is considered as an abnormal outcome.

Our aim was to determine what can be defined as an abnormal outcome (preneuropathy or neuropathy) using all relevant data that can be extracted from the multifrequency vibrometry data and comparing it with the clinical outcome i.e. diabetic foot ulcers and neuropathic symptoms.

## Research design and methods

The local ethics committee at Lund University approved the study (2007/386, 2015/3). Written informed consent was obtained from all participants.

### Study population

All patients at the Department of Endocrinology diagnosed as having type 1 diabetes or latent autoimmune diabetes in adults (LADA) were asked to participate in the study and for their informed consent. The type 1 diagnosis was based on the occurrence of GAD and/or IA-2 antibodies and C-peptide levels at the time of the diagnosis. From May 2015, a total of 535 patients (265 males and 270 females) were recruited. Non-diabetic control subjects were recruited from the same area and up to January 2018 VPTs from 717 (211 males and 506 females) control subjects had been included. To enable comparison with the results in the T1DM population, control subjects were age and gender matched before analysis.

### Patients’ characteristics

Possible symptoms of diabetic neuropathy were evaluated using the modified Toronto Clinical Neuropathy score (mTCNS) [15]. The mTCNS symptoms include foot pain, numbness, tingling, weakness, ataxia and upper limb symptoms. Symptoms are graded from 0 to 3; 0 = symptoms are absent, 1 = present, but no interference with sense of well-being or activities of daily living, 2 = present, interferes with sense of well-being, but not with activities of daily living, 3 = present and interferes with both sense of well-being and activities of daily living (both). After signing an informed consent form the patient, with the help of their doctor, answered a questionnaire about neuropathic symptoms. Year of diagnosis, age, gender, weight, height, blood pressure, HbA_1c_, urinary albumin/creatinine ratio, blood lipids, S-creatinine, presence of macrovascular disease (cardiovascular, cerebrovascular or peripheral vascular disease) and grade of retinopathy were recorded. Macrovascular disease was defined as patients having had angina pectoris, myocardial infarction, cerebrovascular insult or transitory ischemic attack or peripheral vascular disease (according to a physician). Information about ischemic heart disease and cerebrovascular disease is gathered annually and reported to the Swedish National Diabetes Register. Patients diagnosed as having ICD 10 codes I73.9 or E10.5 during the preceding five years were considered to have peripheral arterial disease. The occurrence of diabetic ulcers during the last five years was recorded. The information on diabetic foot ulcers (previous or current) is reported annually in the Swedish National Diabetes Register and was used to identify patients. A diabetic foot ulcer was defined as a foot ulcer of at least Wagner grade 1, that is located below the malleolus. The Department of Endocrinology serves all patients with diabetic foot ulcers in the area and the patient should have received treatment for the ulcer at the diabetic foot clinic for at least 2 weeks.

### Vibration perception thresholds

Vibration perception thresholds were measured at two different sites on the plantar surface of the foot (at the heads of the first and fifth metatarsal bones) with a standard VibroSense Meter (VibroSense Dynamics, Malmö, Sweden) using a method called multifrequency vibrometry. The investigated area is stimulated with a vibrating probe vibrating at six different frequencies (4, 8, 16, 32, 64 and 125 Hz).

Prior to the investigation, the foot temperature was measured using K-type Standard ST-612 thermometer (Taiwan), as temperature may affect the result.

The contact pressure of the probe against the skin was adjusted manually prior to the measurement to a force of approximately 0.2 N, which corresponds to a static skin indent of approximately 1.5 mm. The acceleration of the probe, expressed in decibels (dB), is increased at a speed of 3 dB/s until the subject perceives a vibration, and presses a hand switch. The intensity then decreases at a speed of 3 dB/s until the subject can no longer feel the vibrations, whereupon the subject releases the switch. The procedure is repeated for each frequency. The investigation procedure has been described in detail earlier [12, 17].

VPTs were recorded only on the sole of the right foot in control patients, whereas in diabetic patients both feet were examined. All VPTs on T1DM patients were measured at the Department of Endocrinology by the same person. Non-diabetic control patients were measured in various locations at room temperature with normal humidity by three different investigators. The measuring procedure was similar for cases and controls.

### Statistical analyses

Data on normally distributed values are presented as mean ± standard deviation (SD). Vibration thresholds and diabetes duration were not distributed normally in patients and all VPT values are given as median and 25^th^ and 75^th^ percentile.

The vibration threshold values for the control subjects were distributed normally and dependent on age and gender. To establish age- and gender-specific normal values, non-diabetic controls were divided in five different age groups (18–29, 30–39, 40–49, 50–59 and 60 years or older). Means ± SD for each age group of males and females were calculated and the normal range for each group was defined as a value below the mean + 1.66 x SD. Consequently 5% of the controls had a VPT above the normal range for each frequency.

Because VPTs on MTH 1 and MTH 5 did not differ in control subjects the MTH 1 and MTH 5 values were pooled when age- and gender-specific normal values were calculated. In statistical analyses the VPTs were given as the median between left and right foot for MTH 1 and 5, respectively, except when calculating the number of frequencies above the normal range. The performances of three different models for defining abnormal VPTs were tested using receiver operating characteristic (ROC) analysis. Models were tested regarding diabetic foot ulcers and neuropathic symptoms. In the next step, a logistic regression analysis was used to adjust for factors that could influence the VPTs. To adjust for factors that could influence the risk for diabetic foot ulcers: age, duration, HbA1c, systolic blood pressure, diastolic blood pressure, gender, retinopathy, microalbuminuria, history of macrovascular disease, current smoking and factors that might itself influence VPTs (temperature and height). The same variables were chosen for logistic regression analysis of neuropathic symptoms. All logistic regression analyses were done with forward selection. IBM SPSS Statistics (Statistical Package for the Social Sciences, SPSS Inc., Chicago, Il, USA) version 25 was used for all statistical analyses, except the VFI, for which RStudio Version 1.1.456 (RStudio, Inc., Boston, MA, USA) was used.

## Results

### General characteristics

Table 1 shows the general characteristics of both type 1 diabetic patients and non-diabetic control subjects. There were significantly more women in the control group. The median duration of diabetes among the T1DM patients was 21 [11–34] years. Diabetic patients had higher BMI and foot temperature than non-diabetic control subjects. The difference in BMI was however similar in male patients vs. controls and female patients vs. controls (data not shown). No further information was available for the controls. Both current and previous smoking was more frequent among the T1DM patients compared to non-diabetic controls.

**Table 1:** Characteristics of study subjects.

	T1DM	Controls
N (M/F)	535 (265/270)	717 (211/506)
Age (yrs.)	47.2±16.1	45.2±13.4
Age at Onset (yrs.)	23.5±14.5	N/A
Duration (yrs.)	21 [11–34]	N/A
BMI (kg/m^2^)	25.3±4.0^1^	24.8±4.0
HbA1c (mmol/mol)	62.6±13.3	N/A
Systolic blood pressure (mmHg)	127.1±16.3	N/A
Diastolic blood pressure (mmHg)	72.1±9.5	N/A
Foot temperature (°C)	27.9±5.9^2^	27.1±2.4
Current smoking n (%)	71 (13.3%)^3^	67 (9.5%)
Previous or current smoking n (%)	207 (38.7%)^4^	15 (22.2%)
Retinopathy n (%)	406 (76.5%)	N/A
Sight threatening retinopathy	53 (9.9%)	N/A
Albuminuria/Macroalbuminuria n (%)	60 (11.2%) / 22 (4.1%)	N/A
Ischemic heart disease n (%)	41 (7.7%)	N/A
Cerebrovascular disease n (%)	22 (4.1%)	N/A
Macrovascular disease total n (%)	61 (11.4%)	N/A
Diabetic foot ulcer n (%)	31 (5.8%)	N/A

^1^p = 0.02

^2^p = 0.007

^3^p = 0.045

^4^p = 4x10^-10^. Cases vs. controls.

### Vibration perception thresholds

**Fig 1:**
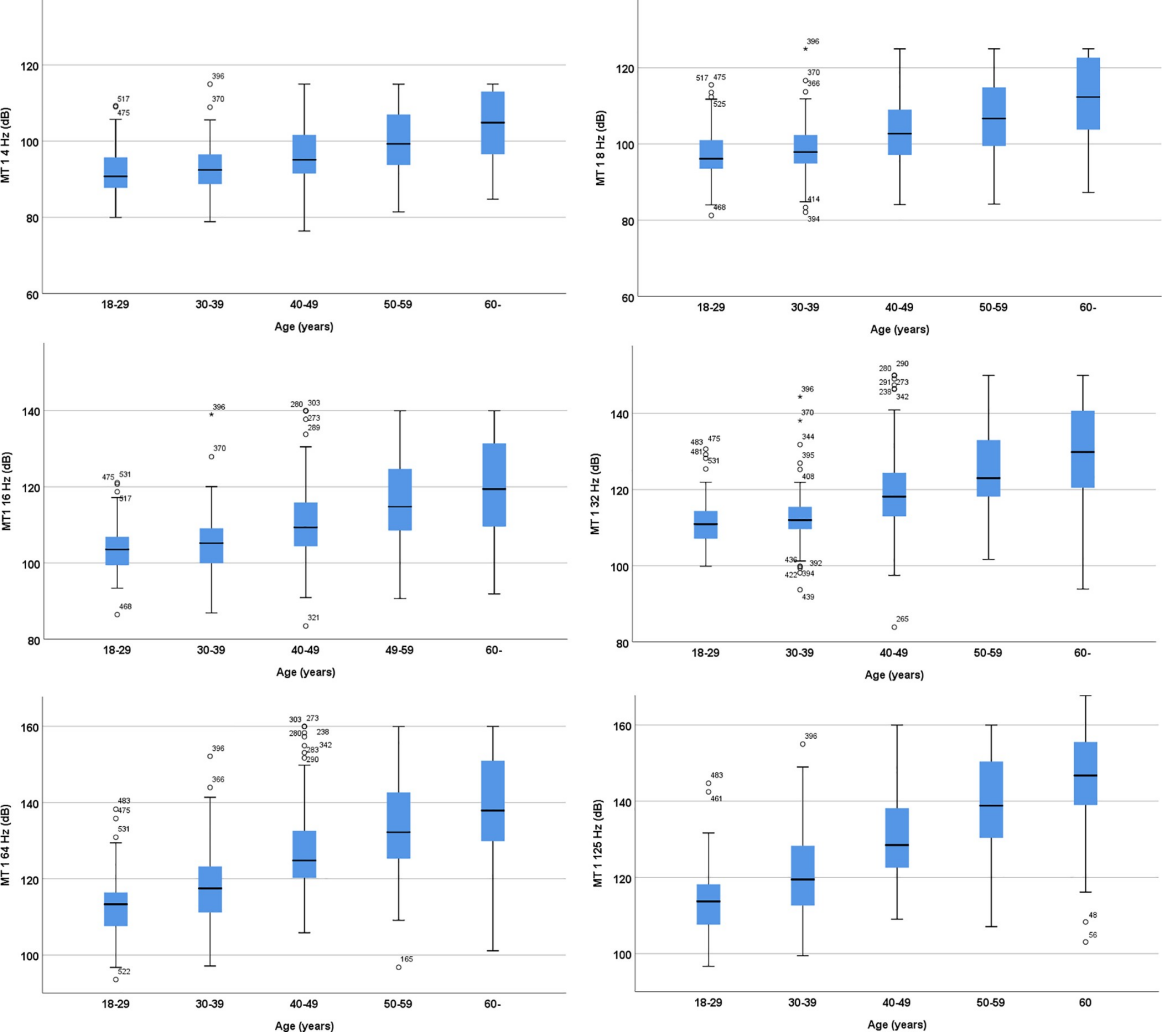
Vibration perception thresholds on the metatarsal head 1 in T1DM patients in different age categories.

Vibration perception thresholds (VPTs) were measured in 717 non-diabetic control subjects 70.6% of whom were women. The VPTs increased with increasing age (Fig 1) and were higher in males than in females (Table 2).

**Table 2:** VPTs at MTH 1 in male vs. female patients.

Frequency (metatarsal 1)	Male	Female
4Hz^a^	98.20[92.25–108.95]	94.75[89.63–100.78]
8Hz^c^	104.43[97.18–119.75]	101.73[95.75–110.72]
16Hz^a^	110.93[105.00–127.95]	108.78 [102.55–117.37]
32Hz^b^	120.14[112.65–136.50]	117.15 [111.34–124.75]
64Hz^a^	128.93[118.02–145.93]	125.07 [114.93–133.77]
125Hz^c^	136.08[119.78–150.62]	129.88 [117.83–142.98]

^a^p< 0.0001

^b^p = 0.001

^c^p = 0.006, male vs female patients. Values are expressed as decibel (dB).

**Fig 2:**
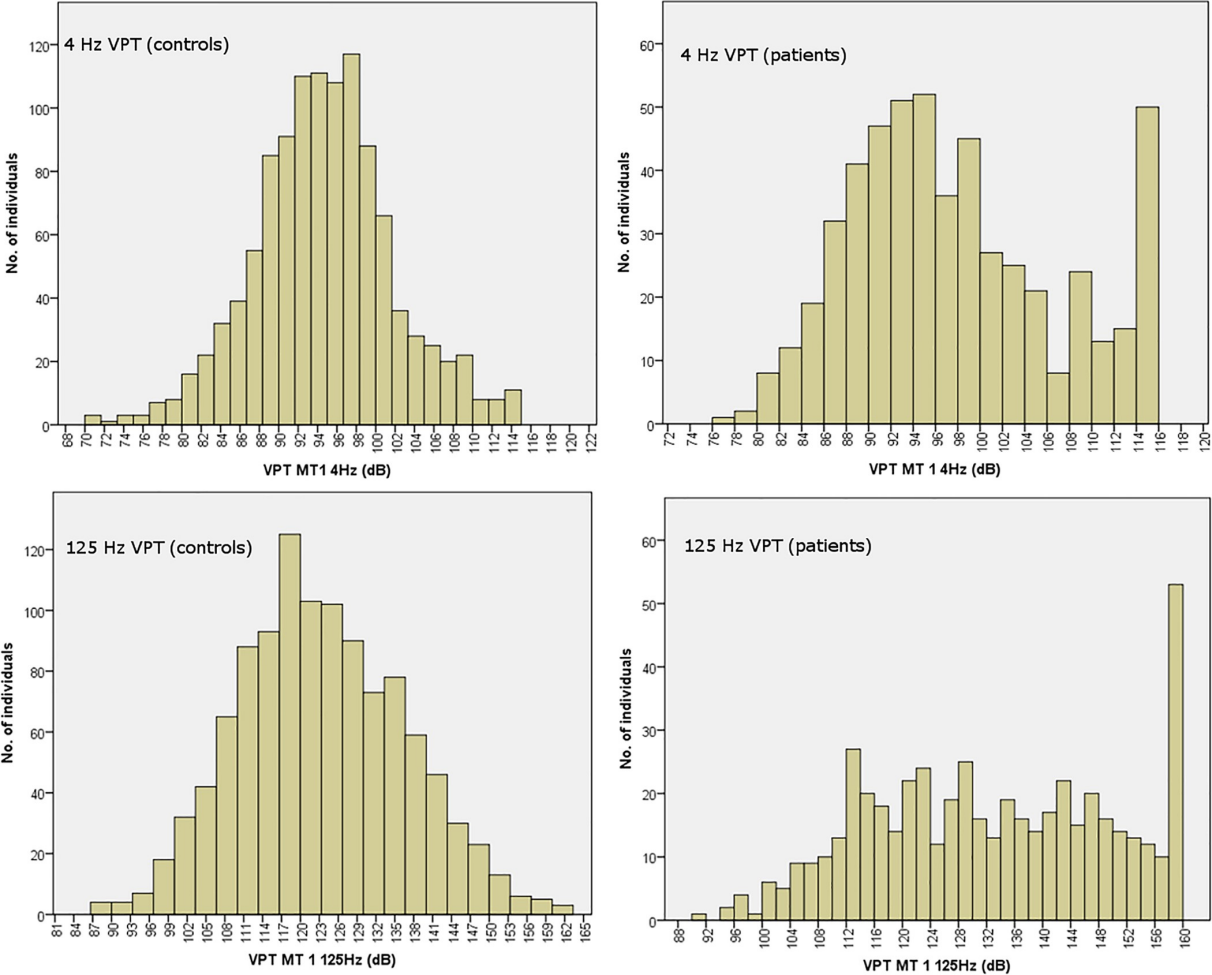
Distribution of VPTs in patients and in controls at 4 Hz and 125 Hz respectively.

The distribution of VPTs in patients and controls (for 4 and 125 Hz, respectively) are shown in Fig 2. A ceiling effect was seen at all frequencies in T1DM patients representing patients, with VPTs higher than the maximum output of the device and thus off the scale. The maximum thresholds were 115, 125, 140, 150, 160 and 170 dB for 4, 8, 16, 32, 64 and 125 Hz, respectively.

The distribution of VPTs at 4 Hz frequency was normally distributed in controls but a bimodal distribution was seen in patients. This bimodal distribution could not be seen at125 Hz frequency, either in patients or in controls (Fig 2).

Table 3 shows the differences between diabetic patients and a subgroup of non-diabetic controls matched for age and gender (N = 498). The VPTs were higher in the diabetic patients than in the non-diabetic controls at all frequencies. When only patients with short-duration patients (≤ 10 years, N = 124) were included, the VPTs at the 125 Hz frequency were higher in patients than in controls (median 120.7 dB vs.118.8 dB, p = 0.008), but no differences were seen at other VPT frequencies.

**Table 3:** Comparison between patients and non-diabetic controls matched for age and gender.

	T1DM	p	Controls	T1DM short duration^a^	Controls	p
Age (yrs.)	45.2±14.8	0.34	45.1±14.1			
Height (cm)	173.6±9.7	0.59	173.8±9.9			
4 Hz (dB)	95.8[90.1–105.5]	<0.0001	92.2[88.6–96.9]	92.9[88.2–98.1]	91.0[88.4–95.7]	NS
8 Hz (dB)	102.0[96.5–112.2]	<0.0001	98.1[94.4–103.6]	98.4[94.4–103.6]	97.8[94.1–101.4]	NS
16 Hz (dB)	109.1[103.5–119.3]	<0.0001	106.4[102.3–111.4]	105.9[101.2–110.4]	105.1[101.8–108.9]	NS
32 Hz (dB)	117.5[111.7–127.9]	<0.0001	113.6[109.7–118.6]	113.6[109.7–117.7]	111.9[108.4–116.3]	NS
64 Hz (dB)	125.3[115.5–136.1]	<0.0001	121.5[114.6–127.9]	116.9[111.8–124.6]	117.5[111.9–125.1]	NS
125 Hz (dB)	130.5[117.8–145.9]	<0.0001	122.7[114.0–134.4]	120.7[113.2–131.3]	118.8[111.6–126.8]	0.008

^a^Duration ≤ 10 yrs. dB = decibel

### Diabetic foot ulcer

#### Roc analysis

**Fig 3:**
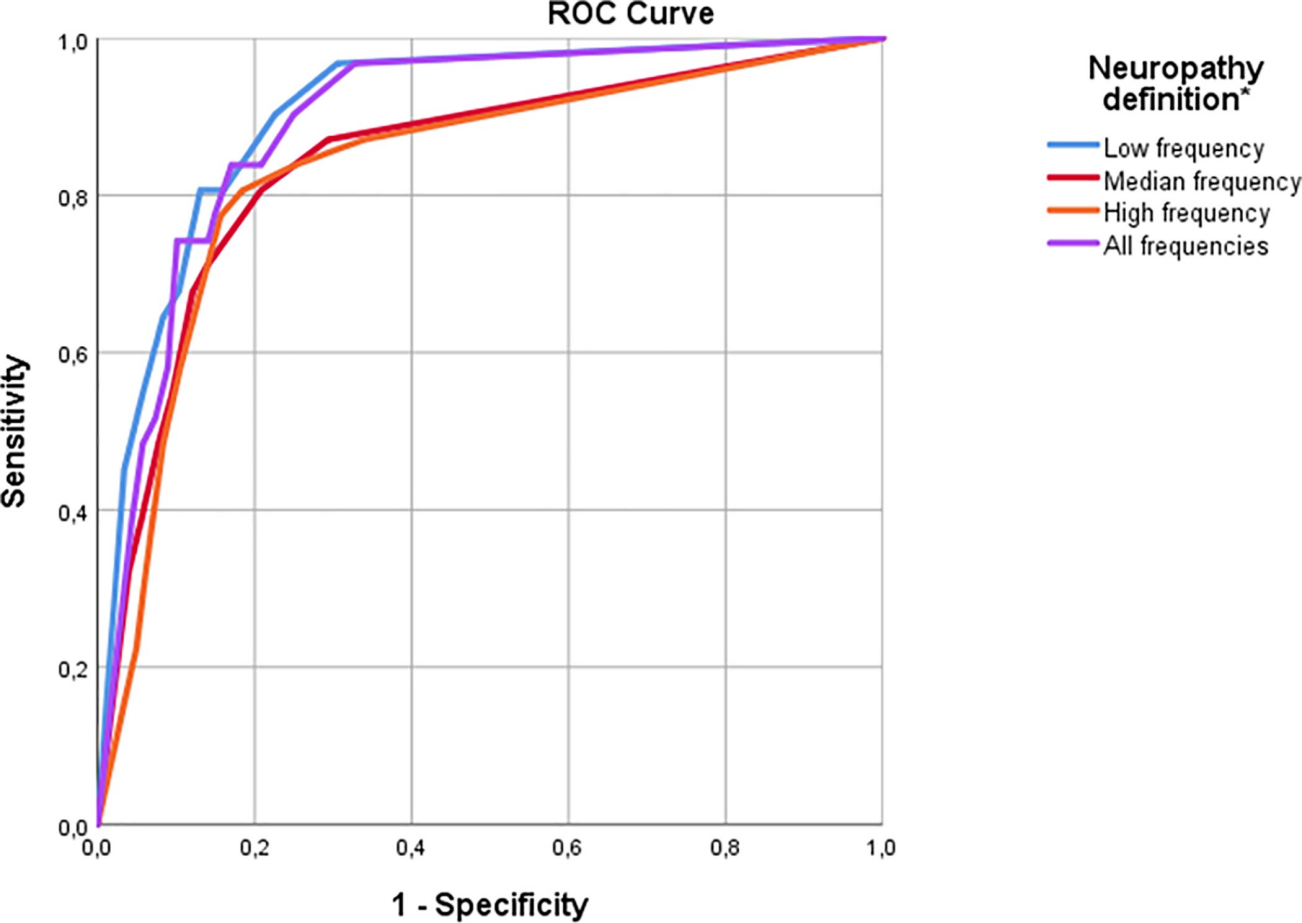
Receiver operating characteristic (ROC) curve of number of VPT frequencies over normal predicting diabetic foot ulcers. Number of low frequencies (4 or 8 Hz) over normal range: AUC = 0.91[0.84-.096], p = 3.1x10-14. Number of median frequencies (16 or 32 Hz) over normal range: AUC = 0.84[0.77–0.92],p = 1.3x10-10. Number of high frequencies (64 or 125 Hz) over normal range: AUC = 0.83[0.76–0.91], p = 4.0x10-10. Number of all frequencies over normal range: AUC = 0.89[0.83–0.94], p = p = 5.3x10-13.

Three different combinations of VPT frequencies were evaluated regarding their relation to a history of foot ulcers: one using all measured frequencies, a second using only the lowest frequencies (4 and 8 Hz) and a third using only high frequencies (64 and 125 Hz), corresponding roughly to the frequency used in the biothesiometer. A ROC analysis for testing foot ulcer was carried out (Fig 3) to determine the optimal number of frequencies over the normal range that predict the risk of developing a diabetic foot ulcer. The median frequencies 16 and 32 Hz did not provide any more information and were therefore excluded from further analysis.

Having at least six or more frequencies (out of a total of 24 measurements) over 1.66 x SD gave the best sensitivity and specificity (90.3% and 82.1%, AUC 0.89 p<0.0001).

The second combination of frequencies included the lowest VPT frequencies 4 Hz and 8 Hz. Having one or more of these frequencies (out of a total of eight) over a normal range had 90.3% sensitivity and 77.4% specificity, AUC 0.91, p<0.0001).

The third combination of frequencies included the highest frequencies (64 and 125 Hz). Having at least one out of a total of 8 measured frequencies over a normal range gave 80.6% sensitivity and 81.5% specificity (AUC 0.83, p<0.0001).

Table 4 shows the frequency of foot ulcer when using different combinations for abnormal VPTs.

**Table 4:** Frequency of foot ulcer with different combinations of abnormal VPTs.

	No	Yes
Six abnormal frequencies	3 (0.7%)	28 (22.0%)
Abnormal Low frequency	1 (0.3%)	30 (16.3%)
Abnormal high frequency	5 (1.3%)	26 (17.0%)

Numbers are N (%). p<1x10^-10^ for all.

#### Regression analysis

To assess the predictive value of different VPTs for the development of diabetic foot ulcer, multiple logistic regression models were performed. Since there was a strong correlation between the VPTs the different frequencies were consequently analyzed in separate regression models. The following covariates were entered into the model to adjust for confounding; foot temperature, age, height, duration, HbA_1c_, systolic blood pressure, diastolic blood pressure, gender, retinopathy, microalbuminuria, history of macrovascular disease, current smoking together with the median value of left and right foot VPT at each frequency respectively. A maximum of 28 cases had missing data. The variance inflation factor (VIF) was less than 2.4 for all combinations of variables. The resulting p-value was multiplied by the number of analysis (twelve). Table 5 shows the results for the regression analysis for different frequencies on MTH 1 and 5, respectively. All studied frequencies were significantly associated with the risk of developing diabetic foot ulcer. The low frequency (4 Hz) on metatarsal head 5 showed the strongest association with a previous diabetic foot ulcer with an OR of 1.24 [1.14–1.35].

**Table 5:** Multinomial logistic regression analysis of variables predictive of diabetic foot ulcer.

	Frequency	Variable	OR	p^a^
MT1	4 Hz	VPT	1.17[1.10–1.25]	0.00004
		Microalbuminuria	4.32[1.77–10.83]	0.02
	8 Hz	VPT	1.15[1.09–1.22]	0.0001
		Microalbuminuria	4.93[1.94–12.53]	0.01
	16 Hz	VPT	1.10[1.06–1.15]	0.00007
		Microalbuminuria	5.67[2.17–14.78]	0.0001
	32 Hz	VPT	1.10[1.05–1.14]	0.0004
		Microalbuminuria	5.15[2.00–13.30]	0.01
	64 Hz	VPT	1.09[1.05–1.13]	0.0001
		Microalbuminuria	5.10[2.06–12.65]	0.005
		Macrovascular disease	3.84[1.53–9.64]	0.048
	125 Hz	VPT	1.10[1.05–1.15]	0.002
		Microalbuminuria	6.21[2.48–15.54]	0.001
		Macrovascular disease	4.74[1.87–11.98]	0.01
	MT5	4 Hz	VPT	1.24[1.14–1.35]	0.000003
			Microalbuminuria	5.54[2.10–41.59]	0.006
		8 Hz	VPT	1.16[1.09–1.23]	0.000006
			Microalbuminuria	6.68[2.58–17.31]	0.001
		16 Hz	VPT	1.11[1.06–1.15]	0.00002
			Microalbuminuria	6.56[2.53–17.01]	0.001
32 Hz		VPT	1.09[1.05–1.13]	0.00007
		Microalbuminuria	5.60[2.26–13.86]	0.002
		Macrovascular disease	4.44[1.77–11.15]	0.02
64 Hz		VPT	1.09[1.05–1.13]	0.00005
		Microalbuminuria	4.79[1.92–11.97]	0.01
125 Hz		VPT	1.06[1.02–1.09]	0.01
		Microalbuminuria	6.17[2.51–15.18]	0.0009
		Macrovascular disease	5.10[2.03–12.83]	0.006

^a^p-values have multiplied with the number of comparisons (twelve).

Using three different combinations of abnormal VPTs as previously described in the regression analysis, including the same covariates, the odds ratios for foot ulcer was 22.2 [6.4–77.2], p = 0.000001, if neuropathy was defined as having at least six frequencies above normal range, 40.7 [5.4–308.4], p = 0.0003 if neuropathy was defined as at least one of the 4 and 8 Hz frequencies above normal and 8.7 [3.1–24.4], p = 0.00004 when neuropathy was defined as at least two of the 64 and 125 Hz frequencies above normal.

### Vibration perception thresholds and neuropathy symptoms

**Fig 4:**
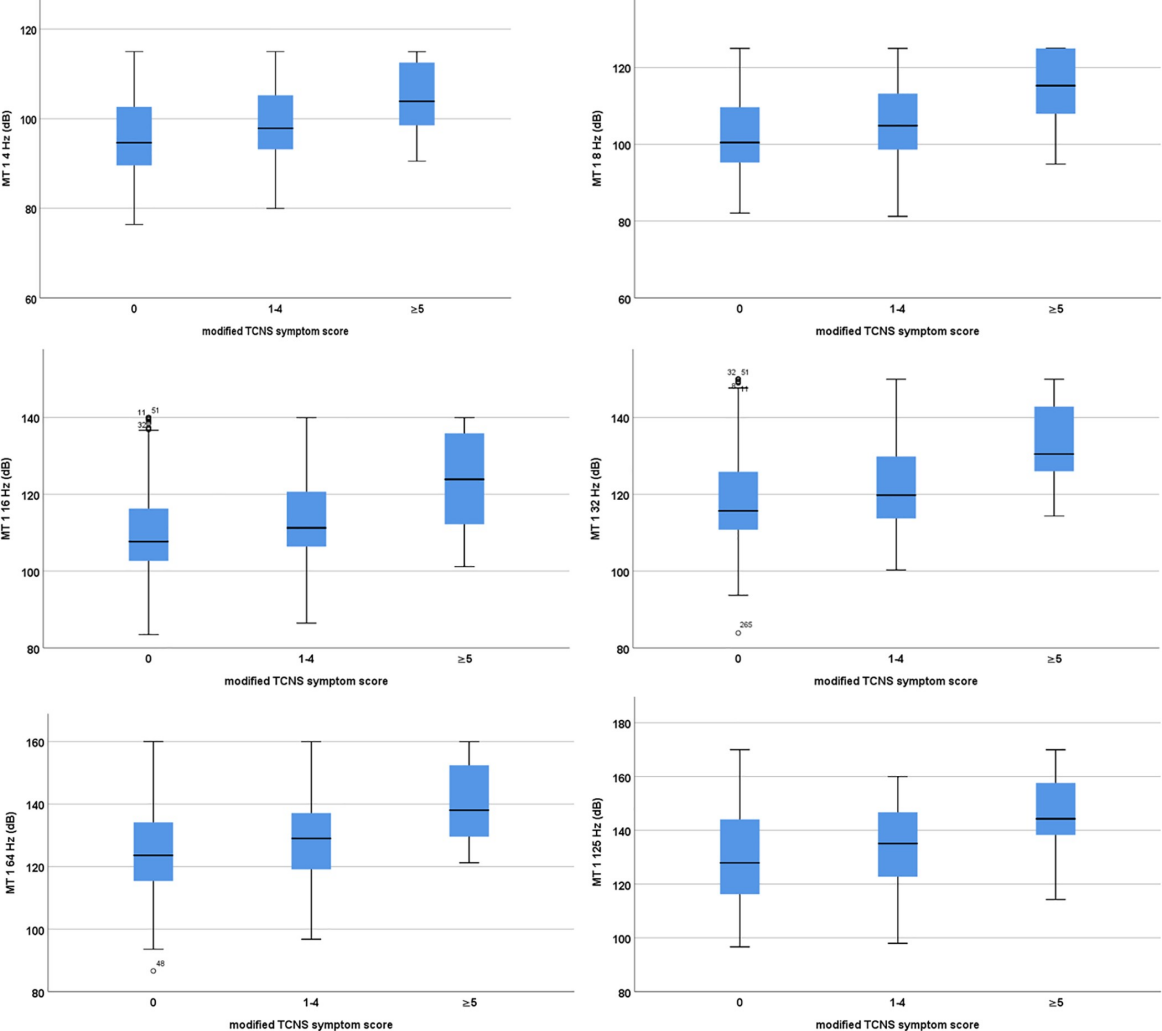
Vibration perception thresholds on the metatarsal head 1 in T1DM patients according to TCNS symptom score.

A higher mTCNS symptom score was associated with higher VTPs on both 4 and 125 Hz (Fig 4). Regardless of for the combination of abnormal VPTs used, there was an association with tingling, weakness and balance/gait problems (the value for upper limb symptoms did not reach a level of significance when corrected for multiple comparisons) (Table 6). Foot pain was associated with having abnormally high VPTs at low frequencies or at the high frequencies.

**Table 6:** Frequency of neuropathic symptoms with three different combinations of abnormal VPTs.

	Six abnormal frequencies^1^			Abnormal low frequency^2^			Abnormal high frequency^3^		
	No	Yes	p-value	No	Yes	p-value	No	Yes	p-value
Foot pain (%)	11.0	15.7	NS	9.7	16.8	0.018	10.2	17.0	0.04
Numbness (%)	16.1	17	NS	15.0	19.0	NS	15.1	19.5	NS
Tingling (%)	14.3	23.6	0.035	12.2	25.2	0.001	12.8	25.8	0.001
Weakness (%)	4.7	15.7	0.0004	3.5	14.6	0.00003	4.7	13.6	0.001
Balance/Gait problems (%)	10.8	23.6	0.002	8.5	24.4	0.000006	10.4	22.7	0.001
Upper limb symptoms (%)	10.8	23.6	NS	13.8	22.4	0.025	14.7	21.9	NS

^1^At least six of the 24 frequencies over normal range

^2^At least one of the 4 Hz or 8 Hz frequencies over normal range

^3^At least two of the 64 or 125 Hz frequencies over normal range

We conducted a logistic regression analysis with self-reported difficulties in walking or balance (or weakness respectively) as the dependent variable and foot temperature, age, gender, height, duration of diabetes, BMI, HbA_1c_, systolic and diastolic blood pressure, microalbuminuria, retinopathy, macrovascular disease and neuropathy (either low or high frequency definition) as independent variables. The variance inflation factor (VIF) was less than 2.4 for all combinations of variables. Having at least one of the lowest frequencies above normal range was associated with a 1.89 fold increase in risk of having problems with balance or gait (OR 1.89 [1.04–3.46], p = 0.04, 64 cases having missing data) and 2.77 fold increase in risk of experiencing weakness (OR 2.77 [1.25–6.16], p = 0.01,67 cases with missing data). In a similar regression analysis having one of the highest frequencies over normal range, was not associated with either gait/balance problems or weakness.

## Discussion

Patients with impaired vibration perception thresholds especially at low frequencies have more often diabetic foot ulcers and neuropathic symptoms than patients with normal VPTs. High frequencies seem to be first affected and VPTs are already impaired in T1DM patients with a disease duration ≤ 10 years compared to non-diabetic controls of a similar age. This this study suggests that it is important to measure vibrotactile sense at both low and high frequencies for better evaluation of the risk for diabetic foot ulcers or symptomatic neuropathy developing in the future.

Similar results have been demonstrated in finger pulps of T1DM patients as the VPTs were increased, particularly at 250 and 500 Hz in both index and little finger pulps, indicating affected median and ulnar nerves, respectively [17]. A recent study on the vibration response of foot-sole cutaneous afferents showed that the rapid-acting type I afferents (RA I corresponding to Meissner’s corpuscles) had the highest impulse per vibratory cycle at low frequencies (3–8 Hz), although responses (>1:1) were seen up to 60 Hz [18]. The rapid-acting type II afferents (RA II corresponding to Pacinian corpuscles) were found to be the class most sensitive to vibration stimuli. The highest response (>3:1) was found at low frequencies, but the response could be seen over the whole frequency range up to 150 Hz at the available vibration amplitudes. The RA II fibers also had the lowest entrainment thresholds, but a larger amplitude was needed at higher frequencies [18]. A similar observation was seen in our current study as higher amplitude was needed at higher frequencies.

There was a strong correlation between VPTs at different frequencies, which could imply that there is an overlap in the response for at least Pacinian corpuscles as previously suggested [18]. Interestingly, the 4 Hz frequency was bimodally distributed in the diabetic patients but not in controls. The bimodal distribution is seen for example in blood glucose distribution and separates individuals with normal glucose tolerance from those with diabetes [19, 20]. This bimodal distribution might therefore indicate that the 4 Hz frequency could be useful in distinguishing between patients with and without neuropathy. However, the bimodal distribution could be due to more patients reaching the upper limit for the amplitude at the 125 Hz frequency than at the 4 Hz frequency. If the amplitude of the frequency at 125 Hz could be increased, one might possibly see a similar peak at this frequency. There were, however, more patients at the 4 Hz frequency (9.5%) that reached the upper limit of the instrument than at 125 Hz frequency (6.5%), suggesting that this is not the case.

It is worth noting that the strongest association between VPTs and diabetic foot ulcer was seen at the 4 Hz frequency at the metatarsal head 5 (Table 5). Mechanoreceptors in the foot are important for posture, balance, standing and walking. Unlike in the hand, the input from Meissner’s corpuscles alone seems to modulate proprioception at the ankle joint in a passive joint-matching task, while the Pacinian input is much less important [21]. Electrical stimulation applied to the sole of the foot during gait could alter lower limb steering [22]. Impaired signaling of the mechanoreceptors could therefore lead to altered plantar pressure and the risk of developing diabetic foot ulcer.

The risk of falling is increased in T2DM patients [3]. We found that the self-reported difficulties in balance or gait were associated with the VPTs at 4 Hz frequency. The current study suggests that the VPTs at the lowest frequencies might be important not only in risk assessment of diabetic foot ulcers, but also in balance and gait difficulties, thus serving as a risk marker for risk of falling in diabetic patients. Although the 125 Hz frequency, which corresponds to the frequency used in a biothesiometer or tuning fork was associated with risk of diabetic foot ulcer, impairment of VPTs at the low frequencies was superior with regard to both the risk of developing diabetic foot ulcer and to problems with gait, balance and weakness.

The ceiling effect for the VibroSense Meter is similar to that which is well known in connection with biothesiometer [23]. We therefore believe that VibroSense Meter should mainly be used for detection of early mild or moderate neuropathy and other simpler tools, such as monofilaments, may be as effective in the detection of severe neuropathy. This study is not a prospective study and thus it cannot answer some important questions, such as whether there is an absolute limit to the VPT after which the neuropathy is irreversible or whether the VPTs could be improved up to a certain limit. With a longer observation time, it might also be possible to establish when the risk of developing diabetic foot problems would increase. One additional limitation to the study might be that a pooled data were used when calculating age- and gender-specific normal values for VPTs. It seems that there is no difference in VPTs regarding location in control subjects whereas the VPTs seem to be first affected at MTH 5 in diabetic patients. A possible source of error could be the fact that measurements were conducted by different people at different localities. The VibroSense Meter has, however, been shown to have a good test-retest reliability [11]. An additional advantage compared to the biothesiometer is that the pressure against skin is tightly regulated. Foot temperature can vary seasonally which could also affect the results. Foot temperature was lower than that previously reported for hand [24]. Unlike in earlier studies concerning the hand, there was no correlation between the foot temperature and vibration thresholds (after correction for multiple comparisons). Given these limitations, this study is still the first to show that, for the purposes of detecting the risk of developing diabetic foot ulcer, low frequencies for measuring vibration-perception thresholds are better than the frequencies normally/currently used. The instrument we used produces six different frequencies and the examination takes30 to 45 minutes in total to perform. Our results suggest that using two low frequencies (4 and 8 Hz) and two higher frequencies (64 and 125 Hz) would be sufficient to detect both the risk of developing diabetic foot problems and early changes in vibration-perception thresholds; i.e. signs of neuropathy.

## Abbreviations

mTCNS: modified Toronto Clinical Neuropathy score
MTH1: Metatarsal head one
MTH5: Metatarsal head five
VPT: Vibration perception threshold
T1DM: Type 1 diabetes
HAVS: Hand-Arm Vibration Syndrome
